# Etymologia

**DOI:** 10.3201/eid1511.999999

**Published:** 2009-11

## *Burkholderia* [burk′′hol-dēr′e-ə]

This genus of gram-negative, rod-shaped bacteria comprising animal and plant pathogens was named for American plant pathologist Walter H. Burkholder. Dr. Burkholder first described a particular species of this genus, later called *Burkholderia cepacia* (Latin for “like onion”), after an outbreak of infection in vegetable growers in New York State in 1949. Previously known to cause disease in onion bulbs, these organisms are now recognized as major bacterial lung pathogens in patients with cystic fibrosis. *B. mallei* causes glanders in horses, and *B. pseudomallei* is the etiologic agent of melioidosis in humans and animals. Dr. Burkholder is recognized for helping establish the role of bacteria as plant pathogens.

